# Spontaneous Tumour Lysis Syndrome in Mantle Cell Lymphoma: A Case Study

**DOI:** 10.7759/cureus.29851

**Published:** 2022-10-03

**Authors:** Nadia Taha, Gopika Bhaskar

**Affiliations:** 1 Haematology, Pinderfields General Hospital, Leeds, GBR; 2 Haematology, Mid Yorkshire Hospitals NHS Trust, Wakefield, GBR

**Keywords:** lymphoma, mantle cell lymphoma, haematology, spontaneous tumour lysis syndrome, tumour lysis syndrome

## Abstract

Tumour lysis syndrome (TLS) is an onco-metabolic emergency seen in rapidly proliferative malignancies resulting from the destruction of tumour cells, resulting in an electrolyte and metabolic derangement. TLS is usually associated with high-grade haematological malignancies and rarely with solid tumours. TLS can be therapy induced or might occur spontaneously. Here, we present a case of a 61-year-old male patient with newly diagnosed mantle cell lymphoma (MCL) admitted for elective chemotherapy, who went into sudden spontaneous tumour lysis before the administration of cytotoxic chemotherapy. The laboratory investigations were consistent with hyperkalaemia, hyperuricaemia, hyperphosphatemia and acute kidney injury. The patient was managed with aggressive intravenous hydration and rasburicase, and his hyperkalaemia was managed in the ward. He was taken to the intensive care unit (ICU) for consideration of haemofiltration. Unfortunately, the patient went into multi-organ failure soon after and died. This case emphasises the need to recognise and treat this complication quickly as it can have fatal consequences. Additionally, it stresses the necessity to vigorously screen patients admitted with malignancy and high tumour burden for TLS, even when they do not receive cytotoxic treatment. TLS management includes adequate hydration, the use of uric acid-lowering therapies and minimisation of potassium intake.

## Introduction

Spontaneous tumour lysis syndrome (STLS) is a life-threatening oncologic emergency that is characterised by a group of metabolic and electrolyte imbalances that occur spontaneously without the introduction of any external factors such as chemotherapy agents [1]. Laboratory tumour lysis syndrome (TLS) may include hyperuricaemia, hyperphosphatemia, hyperkalaemia and hypocalcaemia. These electrolyte disturbances can result in renal failure, cardiac arrhythmias and seizures. These manifestations can lead to multi-organ failure and consequently can be fatal [1,2].

Informed consent was obtained from the patient’s executive of their estate as the patient has expired.

## Case presentation

A 61-year-old male presented to his general practitioner with generalised fatigue after contracting COVID-19 six months ago. He reported a two-month history of unintentional weight loss of one stone with a reduced appetite. He had a dry cough for two months associated with night sweats, insomnia, shortness of breath on exertion and abdominal bloating. His initial blood results showed lymphocytosis (Table 1) comprising mainly mature cells with a proportion of larger pleomorphic nucleolated cells. He had no previous medical history.

**Table 1:** Laboratory results *Abnormal result Hb: haemoglobin; WBC: white blood cell; RBC: red blood cell; MCV: mean corpuscular volume; Hct: haematocrit; MCH: mean corpuscular haemoglobin; MCHC: mean corpuscular haemoglobin concentration; RDW: red cell distribution width; LDH: lactate dehydrogenase

Parameters (normal reference range)	Units	Day 30 (date of death)	Day 1 (first presentation)
Full blood count			
Hb (130-180)	g/L	*57	*109
WBC (4-13)	10^9^/L	*695.8	*154.4
Platelets (135-400)	10^9^/L	246	249
RBC (4.5-6)	10^12^/L	*2.40	*4.45
MCV (80-98)	fL	*100.4	*78.9
HCT (0.40)	1/1	*0.245	*0.351
MCH	Pg	*23.4	*24.5
MCHC	g/L	*233	311
RDW	%	*28.6	*17.5
Nucleated red cells (0-0)	10^9^/L	*4.4	*0.3
LDH (135-250)	u/L	*798	
Liver function test			
Albumin (35-50)	g/L	*34	40
Alkaline phosphatase (30-130)	u/L	*258	*340
Alanine transaminase (0-40)	u/L	36	20
Bilirubin (0-21)	umol/L	18	11
Urea and electrolytes			
Sodium (133-146)	mmol/L	138	139
Potassium (3.5-5.3)	mmol/L	*6.9	4.7
Urea (2.5-7.8)	mmol/L	*15.6	5.3
Creatinine (59-104)	mmol/L	*205	73
Bone profile			
Measured calcium	mmol/L	2.34	2.40
Phosphate (0.80-1.50)	mmol/L	*3.83	0.91
Adjusted calcium (2.20-2.60)	mmol/L	2.54	2.54

The patient was seen by the haematology team within a week of presentation. A plan was made for immunophenotyping, lymphoma blood panels, computed tomography (CT) scan from the neck to the pelvis and discussion in the haematology multidisciplinary team (MDT) meeting with follow-up in one week’s time. On examination, there was widespread lymphadenopathy in the neck, axilla and groin up to 2 cm and a palpable spleen. On auscultation of the chest, there were reduced breath sounds at the lung bases bilaterally.

On flow cytometry, the features on peripheral blood were consistent with mantle cell lymphoma (MCL). Peripheral blood showed involvement with CD5+ B-lymphoproliferative disorder. The phenotype was not typical for chronic lymphocytic leukaemia (CLL). Molecular studies to refine the diagnosis were pending. The sample demonstrated the presence of IGH/CCND1 rearrangement. This was not the blastoid variant. A CT scan of the abdomen and pelvis with contrast showed extensive lymphadenopathy above and below the diaphragm, with huge splenomegaly and focal splenic infarctions and with collapse and consolidation in the left lower lobe and an associated left pleural effusion. The features were consistent with a lymphoproliferative disorder. MDT plan included admitting the patient to the haematology ward for an echocardiogram and with rituximab, cyclophosphamide, hydroxydaunorubicin, oncovin and prednisone regimen (R-CHOP) alternating with rituximab, dexamethasone, cytarabine and cisplatin (R-DHAP) with the intention of proceeding with an autologous stem cell transplant.

On day 20 after presentation, the patient was admitted to the haematology ward as an elective admission to receive cycle 1 of R-CHOP. The patient had not received any steroids prior to commencing treatment. The same evening, he had multiple bouts of vomiting. On assessment, his vital signs were as follows: temperature of 36.3°C, heart rate of 86 beats/minute, respiratory rate of 24 breaths/minute, 99% oxygen saturation on room air and blood pressure of 110/64 mmHg. On general examination, the patient was alert and orientated and reported feeling anxious. The patient was given intravenous fluid hydration. Following further bouts of vomiting only hours later, a venous blood gas demonstrated hyperkalaemia and hyperuricaemia. An electrocardiograph (ECG) showed normal sinus rhythm with peaked T-waves in V2. The patient’s biochemistry results (Table 1) showed a marked lymphocytosis of 695.8 × 10^9^/L and a haemoglobin of 57 g/L. The patient showed hyperkalaemia at 6.9 mmol/L, hyperuricaemia at 15.6 mmol/L and hyperphosphatemia at 3.83 mmol/L with an acute injury stage 2. Spontaneous tumour lysis was identified. An arterial blood gas (Table 2) demonstrated a pH under 6.8 with hyperkalaemia at 9 mmol/L and lactate of over 20 mmol/L. Rasburicase was administered, as well as intravenous fluids and hyperkalaemia treatment. The patient was on strict fluid balance monitoring.

**Table 2:** Arterial blood gas results *Abnormal result pCO_2_: partial pressure of carbon dioxide; pO2: partial pressure of oxygen; tHb: total haemoglobin; Lac: lactate; O2Hb: oxyhaemoglobin; COHb: carboxyhaemoglobin; metHb: methaemoglobin; HHb: deoxyhaemoglobin; sO_2_: oxygen saturation; HCO_3_: bicarbonate

Arterial blood gas (normal reference range)	Units	Result
pH (7.35-7.45)		*<6.80
pCO_2_ (4.6-6.4)	kPa	*3.7
pO_2_ (11-14.4)	kPa	12.5
Na+ (136-145)	mmol/L	*133
K+ (3.5-5.3)	mmol/L	*9
Cl- (95-108)	mmol/L	99
Ca++ (1.15-1.27)	mmol/L	1.25
Glu (2.5-7.8)	mmol/L	*0.5
Lac (0.5-2)	mmol/L	*>20
tHb (115-180)	g/L	*54
O_2_Hb (95-98)	%	*90
COHb (0.5-1.5)	%	*3.1
MetHb (0-1.5)	%	0.8
HHb (0-5)	%	*6.2
sO_2_ (94-98)	%	93.6
BE (B) (-2-3)	mmol/L	*Incalculable
HCO_3_- (C) (22-29)	mmol/L	*Incalculable
HCO_3_- (standard)	mmol/L	*Incalculable

The patient was receiving ongoing treatment for STLS and consented to chemotherapy. The patient was transferred to the intensive care unit (ICU) with the plan for continuous renal replacement therapy (CRRT) and delivery of chemotherapy. On assessment, the patient demonstrated Kussmaul breathing and mild agitation. The severity of TLS was discussed with the patient and family and a ‘do not resuscitate form’ was put in place due to the aggressive nature of the STLS. Within the next few hours, the patient passed away.

## Discussion

Typically, MCL is categorised into a low-risk group for TLS. There is a rare variant of MCL known as the blastoid variant, which is highly aggressive and has a poor prognosis. The patient in this case did not have the blastoid variant. The patient had intrinsic risk factors that predisposed him to developing STLS, including a WBC count of 695.8 × 10^9^/L, massive splenomegaly and an LDH over twice the normal limit [3].

Diagnosis of tumour lysis syndrome

The diagnosis of TLS is defined by the Cairo and Bishop criteria. It defines laboratory and clinical TLS (Table 3). A laboratory diagnosis consists of an abnormality in two or more of the following, occurring within three days before or seven days after chemotherapy or a 25% change from baseline: uric acid, phosphate, potassium or calcium. A clinical diagnosis consists of laboratory TLS with one or more clinical criteria. This system differentiates patients who do not require therapeutic intervention from those experiencing life-threatening complications [4-7].

**Table 3:** Cairo and Bishop diagnostic criteria *May not be directly related to a therapeutic agent TLS: tumour lysis syndrome

Cairo and Bishop diagnostic criteria for TLS (2014) [8]	
Laboratory TLS	Clinical TLS*
Uric acid ≥ 476 mmol/mL (>8 mg/dL) or increase by 25%	Creatinine >1.5 times the upper limit of age-adjusted range level
Phosphorus ≥ 1.45 mmol/L (>4.5 mg/dL) or increase by 25%	Cardiac arrhythmia or sudden death
Potassium ≥ 6 mmol/L (>6 mEq/L) or increase by 25%	Seizure
Calcium ≤ 1.75 mmol/L (<7 mg/dL) or decrease by 25%	

Pathophysiology of spontaneous tumour lysis syndrome

**Figure 1:**
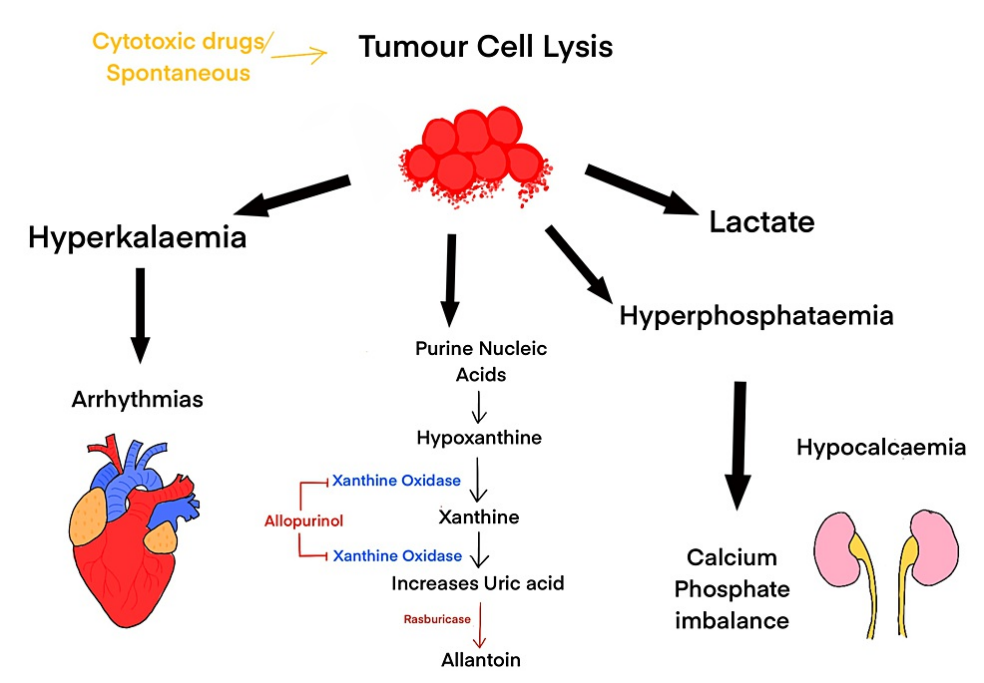
Pathophysiology of TLS Source: Author’s own creation

The exact mechanism of STLS is unknown. Previous studies have suggested that the high formation of glucocorticoids and hyperthermia result in tumour cell lysis [5,9]. There is rapid lysis of malignant tumour cells releasing its metabolites and intracellular ions into the bloodstream [5]. Hyperkalaemia causes arrhythmias, and hyperphosphatemia causes hypocalcaemia, which can lead to seizures, tetany and arrhythmias. This may lead to the deposition of calcium phosphate crystals in organs such as the kidneys, leading to acute kidney injury [10]. The disruption in the body’s mechanism of processing and excreting its metabolites results in the clinical manifestation occurring in TLS [2,4]. Intracellular nucleic acids are catabolised to uric acid through the purine catabolism pathway, resulting in hyperuricaemia [11]. TLS often results in acute kidney injury, and the mechanism of this is multifactorial. Hyperuricaemia causes the deposition of uric acid crystals obstructing the nephron, causing raised tubular pressure [12,13]. Hyperphosphatemia results from the release of intracellular phosphates in the cancer cells, which later precipitate with calcium in the renal tubules, causing acute renal failure [7]. Hypocalcaemia in TLS results from this precipitation and can lead to tetany and cardiac arrhythmias [4]. Studies have shown that patients with STLS may have lower rates of hyperphosphatemia due to phosphate uptake into rapidly dividing tumour cells [14]. Hyperkalaemia is one of the key laboratory findings of TLS, which occurs due to the kidneys’ inability to process the excess released potassium. Increased serum concentrations of potassium can adversely affect the skeletal muscle and cardiac myocardium, leading to ventricular tachyarrhythmias and even death [4,14]. The pathophysiology of TLS is demonstrated in Figure 1.

Risk stratification

TLS is most associated with haematological malignancies including acute leukaemia and non-Hodgkin lymphoma. There is a 4%-42% incidence of TLS in the treatment of these malignancies [15]. Although TLS is more commonly seen after cytotoxic therapies, STLS is reported typically amongst high-grade haematological malignancies such as B-cell non-Hodgkin lymphoma. Although it is rare, STLS can occur in patients with solid malignancies including gastric, lung and breast cancers. It is difficult to determine the prevalence of this as is it based solely on case reports [1]. The risk for TLS is based on two factors as suggested by most studies. Firstly, the risk is associated with the malignancy itself. The size of the tumour mass, multi-organ involvement, the proliferative potential of the tumour and chemo-sensitivity pose a risk for tumour lysis. Highly proliferative malignancies such as Burkitt’s lymphoma and acute lymphoblastic lymphoma are more at risk for tumour lysis. Secondly, patient factors such as advanced age, hydration status, renal failure, diabetes mellitus and cardiac disease can affect the development of TLS [1,16]. In a retrospective analysis, it was found that 1.08% of patients with haematological malignancies and acute renal failure developed TLS [1].

The risk of TLS can be categorised into low, intermediate or high risk as per a study conducted by Cairo et al. [6]. Low risk involves all solid tumours; unless they are bulky or chemo-sensitive, they are of intermediate risk. Intermediate risk includes acute leukaemia and aggressive lymphoma such as Burkitt’s lymphoma. These are considered high risk if they are bulky or if there is leucocytosis or renal involvement [1,16].

Prevention is better than cure

The best management of TLS is prevention. Patients at low and intermediate risk should be closely monitored and receive hydration and allopurinol. High-risk patients are treated with rasburicase, which reduces the level of uric acid in the blood by converting it to a more stable form that can be excreted more readily [1,15]. There is a lack of evidence for the clinical benefit of rasburicase over allopurinol [7].

The mainstay of prevention is hydration and diuresis, which helps in the excretion of excess metabolites and electrolytes. Patients should receive anywhere between two and four times daily fluid maintenance and maintain a urine output of at least 100-150 mL/hour unless contraindicated for fluid therapy or diuresis. The traditional way of urinary alkalisation is considered controversial these days as urinary alkalinisation with sodium bicarbonate may lead to metabolic alkalosis and/or xanthine obstructive uropathies. The solubility of xanthine and hypoxanthine significantly decreases at an alkaline urine pH (≥6.5), leading to the development of urinary xanthine crystals during and after allopurinol therapy [8].

The second line of prevention is hypouricemic agents such as allopurinol and rasburicase. Allopurinol is a competitive inhibitor of xanthine oxidase and prevents the formation of uric acid (Figure 1). Allopurinol can be administered orally 24-48 hours before the initiation of chemotherapeutic drugs [8]. The major disadvantage of allopurinol is its delayed onset of action and that it does not reduce the amount of urate already present in the body. A substitute to allopurinol is rasburicase, which rapidly brings down urate levels. Rasburicase is recombinant uric acid oxidase cloned from *Aspergillus flavus*. It converts uric acid to allantoin, which is more soluble in urine and is easily excreted. It should be avoided in patients with glucose-6-phosphate dehydrogenase (G-6PD) deficiency [4]. In addition to this, the patient’s daily weight, vital signs, fluid balance and blood sampling (urea, creatinine, potassium, calcium, phosphorous, magnesium, uric acid, LDH and coagulation) need to be closely monitored. Reviewing the medication chart for any nephrotoxic medications is also of the utmost importance [6].

Management

The foundation in the management of established TLS is hydration, rasburicase and tackling the deranged electrolytes and their clinical manifestation. Aggressive fluid therapy to wash out the by-products of tumour cell lysis is the first line of action, as well as the initiation of IV rasburicase at the dose of 0.2 mg/kg once daily. Hyperphosphatemia is difficult to treat if not controlled by hydration and rasburicase. Significantly high levels of phosphate are an indication for renal replacement therapy [14,17]. Symptomatic hypocalcaemia is managed with calcium gluconate in the standard dose for adults and children with continuous cardiac monitoring. Asymptomatic hypocalcaemia should not be treated as it can further precipitate calcium phosphate deposition in the kidneys [17]. Hyperkalaemia is potentially the most fatal abnormality in TLS. Continuous cardiac monitoring and regular blood sampling for electrolyte measurement should be carried out. Hyperkalaemia can be treated as per the established protocols with intravenous infusion of insulin, glucose and calcium gluconate. Nebulised or intravenous salbutamol is also effective [17]. The combination of refractory hyperkalaemia, oliguria, central nervous system (CNS) toxicity secondary to uraemia, metabolic acidosis and fluid overload unresponsive to diuretics is an indication for haemodialysis. TLS is also an indication for haemodialysis in isolation [8,17].

## Conclusions

After reviewing the literature on STLS in mantle cell lymphoma, we believe that only a few cases have been reported so far, and hence, the objective of this case presentation is to alert physicians on the importance of risk assessment. This patient was not receiving prophylactic treatment until he became symptomatic with vomiting and was given intravenous fluid hydration and rasburicase. This case highlights the significance of STLS and the importance of early recognition of haematological malignancies and the commencement of prophylaxis treatment with hydration and monitoring of electrolytes.
